# Tele-transitions of care (TTOC): a 12-month, randomized controlled trial evaluating the use of Telehealth to achieve triple aim objectives

**DOI:** 10.1186/s12875-020-1094-5

**Published:** 2020-02-07

**Authors:** Kimberly Noel, Catherine Messina, Wei Hou, Elinor Schoenfeld, Gerald Kelly

**Affiliations:** Department of Family, Population and Preventive Medicine, Stony Brook Medicine, Stony Brook, New York, 11794 USA

**Keywords:** Telehealth, Readmissions, Transitions of care, Video visits, Remote patient monitoring

## Abstract

**Background:**

Poor transitions of care leads to increased health costs, over-utilization of emergency room departments, increased re-hospitalizations and causes poor patient experiences and outcomes. This study evaluated Telehealth feasibility in improving transitions of care.

**Methods:**

This is a 12-month randomized controlled trial, evaluating the use of telehealth (remote patient monitoring and video visits) versus standard transitions of care with the primary outcomes of hospital readmission and emergency department utilization and secondary outcomes of access to care, medication management and adherence and patient engagement. Electronic Medical Record data, Health Information Exchange data and phone survey data was collected. Multi-variable logistic regression models were created to evaluate the effect of Telehealth on hospital readmission, emergency department utilization, medication adherence. Chi-square tests or Fisher’s exact tests were used to compare the percentages of categorical variables between the Telehealth and control groups. T tests or Wilcoxon rank sum tests were used to compared means and medians between the two randomized groups.

**Results:**

The study conducted between June 2017 and 2018, included 102 patients. Compared with the standard of care, Telehealth patients were more likely to have medicine reconciliation (*p* = 0.013) and were 7 times more likely to adhere to medication than the control group (*p* = 0.03). Telehealth patients exhibited enthusiasm (*p* = 0.0001), and confidence that Telehealth could improve their healthcare (p = 0.0001). Telehealth showed no statistical significance on emergency department utilization (*p* = 0.691) nor for readmissions (*p* = 0.31). 100% of Telehealth patients found the intervention to be valuable, 98% if given the opportunity, reported they would continue using telehealth to manage their healthcare needs, and 94% reported that the remote patient monitoring technology was useful.

**Conclusions:**

Telehealth can improve transitions of care after hospital discharge improving patient engagement and adherence to medications. Although this study was unable to show the effect of Telehealth on reduced healthcare utilization, more research needs to be done in order to understand the true impact of Telehealth on preventing avoidable hospital readmission and emergency department visits.

**Trial registration:**

ClinicalTrials.Gov ID: NCT03528850 Date Registered (Retrospective): 5/18/2018.

**Status:** Completed.

**IRB #:** 970227.

## Background

Telehealth has the potential to improve transitions of care, through enhanced connections among patients and their clinicians, during a vulnerable period after hospital discharge [1]. To achieve triple aim objectives, reducing unnecessary hospital readmissions is desirable for payers and patients alike [2]. Several key studies have shown the values of telehealth in reducing avoidable hospital readmissions [1, 3–7], while others have reported inconsistent findings in regards to overall healthcare utilization: Emergency Department (ED) visits and readmissions [4, 5, 8–11]. Telehealth interventions using primarily communications and surveillance technologies, show most promise in counseling and enhancing patient compliance [12]. It is unlikely that telehealth alone, can reverse disease pathology or predictable courses of disease [13]. Despite this, the majority of published telehealth studies, have focused on patient populations selected by diagnosis, such as heart failure, with limited generalizability regarding the effects of Telehealth in regards to patients with multiple co-morbidities [12]. This study’s main inclusion criteria, rather than initial admission diagnosis, is the patient’s disposition to the home while managing multi-comorbid disease.

Patients in this trial, have an existing primary care provider (PCP) within our health system and received telehealth services from either their PCP or a clinical trainee reporting to their PCP. Patients were enrolled in the study at the bedside, prior to hospital discharge to their homes. The intervention’s primary endpoints were in attempts to reduce hospital readmissions and ED utilization with an overall aim of reducing adverse events through improved patient–provider communication, medicine reconciliation, patient education, and assurance of patient hemodynamic stability. The Telehealth Transitions of Care intervention, or TTOC, was designed in concordance with the Care Transitions Intervention, and Eric Coleman’s four pillars of transitional care, known to be effective in readmission reduction: 1) medication self-management 2) clinical follow up 3) knowledge of clinical “red flags” and 4) increased access to patient-centered documentation [14–18]. TTOC follows the strength of evidence of maximal patient benefit for patients with mixed chronic conditions, by use of a multi-functional approach (remote patient monitoring and video visits) [5, 12]. TTOC was designed to enhance PCP services, while also providing training opportunities for physicians in Telehealth. In doing so, TTOC provided a major benefit to our academic hospital system, helping to overcome known barriers to telehealth adoption [19–21]. We introduce a feasible, replicable approach using clinical trainees and direct involvement of the patient’s PCP. The protocol and study design has been published in the peer reviewed literature [22].

## Methods

### Aim, design and setting of the study

The aim of the study was to evaluate the effects of TTOC (weekly video visits with daily remote patient monitoring), to standard of care. The methods and study design have been previously published and are adherent to the CONSORT Statement guidelines [22, 23]. The primary outcome measures of the study are hospital readmissions and ED visits within 30 days of the index hospitalization discharge (Table 3). Secondary outcome measures include, patient experience (Table 2), medication adherence and management, mortality, and access to care (days to PCP follow up) (Table 3)”. This study was performed by the Family and Internal Medicine Departments at Stony Brook Medicine, which is a 603-bed teaching institution on the northern part of Long Island, New York. The hospital is located in Suffolk County with an annual admission of 31,715 patients. Over 75% of patients were serviced by the Family Medicine physicians, in an ambulatory practice serving about 32,000 patients annually, who do not currently serve uninsured patients (whom are referred to our affiliated Federally Qualified Health Centers and our free student run clinic not officially part of the Family Medicine practice).

### Participant characteristics

**Fig. 1:**
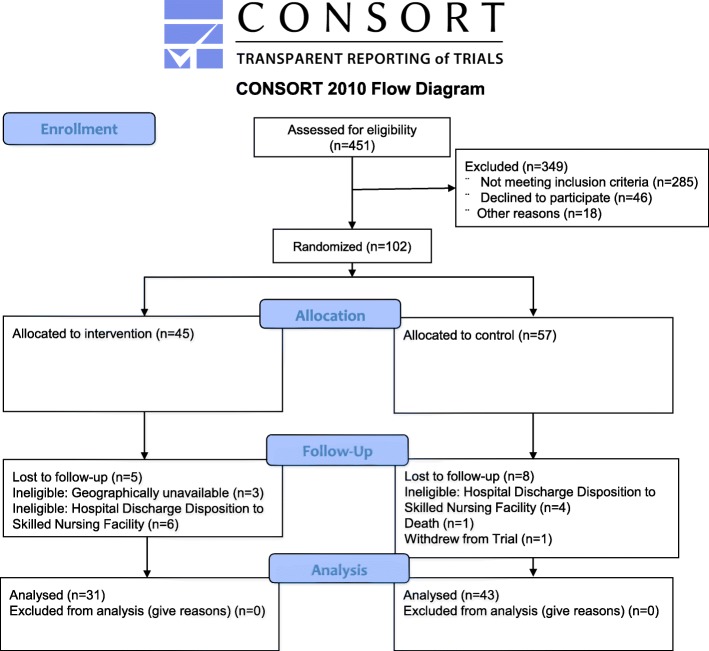
Total CONSORT Statement Flow Diagram

The sample consisted of 102 patients who fulfilled the eligibility criteria, were randomized to receive either TTOC or Standard of Care (Fig. 1). Randomization was conducted using a computer-generated 1:1 random number allocation sequence through REDCap [22]. Eligible patients were adult patients (≥ 30 years), with 2 or more chronic disease processes, English speakers, with good cognitive function, a life expectancy greater than 6 months, with an ability to provide consent. All patients were hospitalized at Stony Brook University Hospital and discharged to the patient home, with the follow up care in either the Family or Internal Medicine clinical practices. Patients self-identified as living within reasonable commute to the Family or Internal Medical Group clinics and were able to complete a technological aptitude test of turning on the telehealth technology and following the prompts. Patients were excluded if they had physical limitations prohibiting the use of the telehealth equipment, were uninsured (who received referrals elsewhere for follow up care), if involved in another research study, were pregnant or actively trying to conceive, or if admitted for a primary psychiatric diagnosis.

### Study design

We conducted a 2-arm, parallel group, randomized controlled trial between June 1, 2017 to June 1, 2018. The standard of care for discharge planning, includes reviewing patient instructions and the discharge summary. The patient is encouraged to follow up with the PCP within 7–14 days and with scheduled specialist appointments as indicated [22]. The Telehealth intervention involved the provision of a telehealth kit, which included a smart phone device a Bluetooth-enabled blood pressure monitoring cuff, pulse oximeter, weighing scale, within 2 days after hospital discharge [22]. Telehealth patients measured their vitals daily using the tele-equipment and had weekly virtual visits with a transition of care physician (teledoc). During visits with the teledoc, medicine reconciliation was documented and medication adherence assessed in concordance with best practice from published studies [24] Physicians were trained to ask open-ended questions to assess medications taken, as well as perform validation of patient histories using Electronic Medical Record (EMR) and pharmacy data. Clinicians documented adherence if there were no discrepancies between physician prescription and patient self-report and data. Upon consent, patients participated in the trial for the length of thirty days following hospital discharge. All patients were consented and enrolled in the Health Information Exchange.

### Data collection

Study data was collected and managed using REDCap [25] electronic data capture tools hosted at Stony Brook Medicine. Primary Outcomes of hospital readmission and ED utilization were assessed using Health Information Exchange and EMR data. Secondary outcomes were assessed through phone surveys, whose data was hosted on REDCap. Medication adherence and reconciliation data was collected in REDCap during the virtual visit. For the control group, the EMR medicine reconciliation records and clinical notes were reviewed by a physician, for documented non-adherence to the discharge summary treatment plan.

### Data analysis

Frequencies and percentages were calculated for categorical variables, e.g. Re-admission and ED visit for each randomized group. Means and standard deviations (SD) were calculated for continuous variables if the data followed a normal distribution (e.g. age). If the data did not follow the normal distribution, medians and ranges were calculated instead. Chi-square tests or Fisher’s exact tests were used to compare the percentages of categorical variables between the Telehealth and control groups. T tests or Wilcoxon rank sum tests were used to compared means and medians between the two randomized groups. To evaluate the associations between Readmission, ED visit and adhere with telehealth and other factors, multivariable logistic regression models were performed, and odds ratios and their 95% confidence intervals were estimated.

SAS v9.3 (Cary, NC) and SPSS v25 (Chicago, IL) were used to conduct all statistical analyses.

## Results

451 patients were assessed for eligibility for the trial. 102 patients met inclusion criteria for study participation, gave informed consent, and were enrolled in the study prior to discharge. 45 patients were randomized to the TTOC group while 57 patients received the standard of care. Patients were excluded if they no longer met the criteria of being discharged to home; if their hospitalization course worsened, requiring discharge to a subacute rehabilitation center (SAR). A small proportion of patients later refused to participate in the trial when discharged to the home (Fig. 1).

Overall, the study arms were balanced by characteristics as there were no statistically significant differences between the groups in regards to demographics including gender, race and education (Table 1). The average age at enrollment was 65 years. 68.5% of individuals in the control group had higher than high-school level of education in comparison to 76.1% of individuals in the telehealth intervention group. In addition, the employment rate was similar in both groups: 43.1% in the control group and 38.3% in the intervention group. No significant difference was seen in the presence of a computer at home: 77.6% for the control group and 63.8% for the telehealth intervention group. Over 97% of study participants had internet in their home, while over 94% owned a cell phone. Both groups had high percentages of patients who used a computer on a regular basis 63.8% in the control group and 77.6% of the TTOC group. Both groups showed high confidence in using smart phones or tablets. On average both groups spent about 4 h on the computer per day. Similar percentage of individuals in both groups reported their health as either good or very good: 60.3% in the control group and 48.9% in the intervention group. The median scores, on a questionnaire scale of 1–10, were also similar between the two groups for the confidence with health management and comfort with technology measures. Patients had an average of nine diagnoses, and a moderate severity risk score and a moderate rating of disability.

**Table 1:** Baseline Demographics

Basic Demographics	Standard of Care *n* = 57	Treatment *n* = 45	*P* value
Age, Mean(SD) ^a^	63.67 (14.78)	65.66 (13.24)	0.483
Female, n (%) ^b^	35 (63%)	29 (64%)	0.840
Education, n (%) ^b^			
High School	16 (30%)	11 (25%)	0.807
Some College	21 (40%)	20 (45%)	
Completed College	16 (30%)	13 (30%)	
Race, n (% Non-Caucasian) ^a^	10 (18%)	6 (13%)	0.561
Employed n (%) ^b^	25 (44%)	17 (38%)	0.535
Sick/Disability			
Readmission Risk Score ^a, d^	45.29 (11.82)	45.28 (14.28)	0.997
Number of Diagnoses, Median ^c^	8	9	0.401
Median Number of Follow Up Appointments on Discharge ^c^	3	2	0.348
How would you rate your health? n (% Good/Very Good) ^b^ (0 = Good/Very Good, 1 = Poor/Fair)	34 (60%)	22 (49%)	0.278
General Health Rating, Median ^c^ (Poor =0, Fair =1, Good = 2, Very Good = 3, Excellent =4)	2 (0,4)	1 (0,4)	0.141
Access to Care			
Emergency Contact Person, Yes n (%) ^b^	57 (100%)	42 (93%)	0.083
Full-time Caregiver, Yes n (%) ^b^	5 (9%)	7 (16%)	0.291
Self-Efficacy			
Confidence in Health Management (0–10: Least Confident-Most Confident, respectively) Median ^c^	9 (4,10)	8 (2,10)	0.146
Computer/Tech Savviness			
Do you use a computer on a regular basis? Yes n (%) ^b^	45 (79%)	29 (64%)	0.103
How comfortable are you with using technology like a smart phone or tablet? (0–10: Least Comfortable- Most Comfortable, respectively) Median (Range) ^c^	8 (0,10)	7 (0,10)	0.225
Do you own a cell phone? ^b^ Yes n (%)	54 (95%)	43 (96%)	0.999
Do you have internet service in your home? Yes n (%) ^b^	56 (98%)	44 (98%)	0.999
Do you have difficulties with your cell service, whereby you experience dropped calls or poor reception? Yes n (%) ^b^	2 (4%)	2 (4%)	0.999
How many hours per day do you use the computer? Mean (SD) ^a^	3.57 (2.90)	4.95 (5.25)	0.154
Telehealth			
How enthusiastic are you about the Telehealth program, (0–10, Least Enthusiastic-Most Enthusiastic, respectively) Median ^c^	8 (0,10)	9 (1,10)	0.124
How confident are you that Telehealth may help your healthcare, (0–10: Least Confident-Most Confident, respectively) Median ^c^	9 (0,10)	8 (3,10)	0.970

^a^ based on t-tests comparing difference in means. The data shows mean (SD) in each randomized group

^b^ based on Chi-square or Fisher’s exact test comparing difference in %. The data shows n (%) in each randomized group

^c^ based on Wilcoxon rank sum tests comparing medians. The data shows median (min, max) in each randomized group

^d^ Risk scores are calculated by using a proprietary algorithm by Cerner© that includes about 40 + data points from groups, based on the patient history and admitting physical exam, diagnosis related group codes, patient demographics, procedures, utilization, lab tests, medications, and exploratory variables. The score uses a scale (0–100 scale) that it easier for clinicians to understand

Patients had a statistically significant improvement in enthusiasm and confidence that Telehealth helped patients (*p* = 0.0001). There was no statistically significant difference in the perception of difficulty in participating in the trial (*p* > 0.072).

Telehealth has the potential to improve transitions of care, through enhanced connections among patients and their clinicians, during a vulnerable period after hospital discharge [1]. To achieve triple aim objectives, reducing unnecessary hospital readmissions is desirable for payers and patients alike [2]. Several key studies have shown the values of telehealth in reducing avoidable hospital readmissions [1, 3–7], while others have reported inconsistent findings in regards to overall healthcare utilization: Emergency Department (ED) visits and readmissions [4, 5, 8–11]. Telehealth interventions using primarily communications and surveillance technologies, show most promise in counseling and enhancing patient compliance [12]. It is unlikely that telehealth alone, can reverse disease pathology or predictable courses of disease [13]. Despite this, the majority of published telehealth studies, have focused on patient populations selected by diagnosis, such as heart failure, with limited generalizability regarding the effects of Telehealth in regards to patients with multiple co-morbidities [12]. This study’s main inclusion criteria, rather than initial admission diagnosis, is the patient’s disposition to the home while managing multi-comorbid disease.

Patients in this trial, have an existing primary care provider (PCP) within our health system and received telehealth services from either their PCP or a clinical trainee reporting to their PCP. Patients were enrolled in the study at the bedside, prior to hospital discharge to their homes. The intervention’s primary endpoints were in attempts to reduce hospital readmissions and ED utilization with an overall aim of reducing adverse events through improved patient–provider communication, medicine reconciliation, patient education, and assurance of patient hemodynamic stability. The Telehealth Transitions of Care intervention, or TTOC, was designed in concordance with the Care Transitions Intervention, and Eric Coleman’s four pillars of transitional care, known to be effective in readmission reduction: 1) medication self-management 2) clinical follow up 3) knowledge of clinical “red flags” and 4) increased access to patient-centered documentation [14–18]. TTOC follows the strength of evidence of maximal patient benefit for patients with mixed chronic conditions, by use of a multi-functional approach (remote patient monitoring and video visits) [5, 12]. TTOC was designed to enhance PCP services, while also providing training opportunities for physicians in Telehealth. In doing so, TTOC provided a major benefit to our academic hospital system, helping to overcome known barriers to telehealth adoption [19–21]. We introduce a feasible, replicable approach using clinical trainees and direct involvement of the patient’s PCP. The protocol and study design has been published in the peer reviewed literature [22].

The aim of the study was to evaluate the effects of TTOC (weekly video visits with daily remote patient monitoring), to standard of care. The methods and study design have been previously published and are adherent to the CONSORT Statement guidelines [22, 23]. The primary outcome measures of the study are hospital readmissions and ED visits within 30 days of the index hospitalization discharge (Table 3). Secondary outcome measures include, patient experience (Table 2), medication adherence and management, mortality, and access to care (days to PCP follow up) (Table 3)”. This study was performed by the Family and Internal Medicine Departments at Stony Brook Medicine, which is a 603-bed teaching institution on the northern part of Long Island, New York. The hospital is located in Suffolk County with an annual admission of 31,715 patients. Over 75% of patients were serviced by the Family Medicine physicians, in an ambulatory practice serving about 32,000 patients annually, who do not currently serve uninsured patients (whom are referred to our affiliated Federally Qualified Health Centers and our free student run clinic not officially part of the Family Medicine practice).

**Table 2:** Patient Experience at 30 Days Post-Hospitalization

	Standard of Care *n* = 43	Treatment 30 day *n* = 31	*P* Value
How Difficult was participation in the Study for you? ^a^ n (%) (0 = Very Easy 4 = Very Difficult)	0 (0,2)	0 (0,2)	0.072
How enthusiastic are you about the Telehealth program? ^a^ (0–10: Least Enthusiastic-Most Enthusiastic, respectively) Median (Range)	7 (0,10)	10 (5,10)	< 0.0001*
How confident are you that Telehealth may help your healthcare? ^a^ (0–10: Least Confident-Most Confident, respectively) Median (Range)	7.5 (0,10)	9 (5,10)	< 0.0001*
How confident are you with managing your own healthcare? ^a^ (0–10: Least Confident-Most Confident, respectively) Median (Range)	9 (1,10)	9 (5,10)	0.914

^a^based on Wilcoxon rank sum tests comparing medians

*denotes statistical significance

**Table 3:** Clinical Endpoints for Telehealth

Effect	Point Estimate	95% Wald Confidence Limits		*P* value
ED Utilization ^a^	0.749	0.180	3.115	0.691
Readmission ^a^	2.645	0.404	17.328	0.311
Medication Adherence ^a^	6.925	1.203	39.856	0.030^c^
	Standard of Care *n* = 57	Treatment *n* = 45	*P* value	Standard of Care *n* = 57
Medicine Reconciliation ^b^	47 (82%)	31 (100%)	0.013^c^	Medicine Reconciliation ^b^
PCP Follow-up Visit, Yes n(%) ^b^	31 (60%)	34 (76%)	0.096	PCP Follow-up Visit, Yes n(%) ^b^
Death ^b^	1 (2%)	0 (0%)	0.372	Death ^b^

^a^based on logistic regression controlling for age, gender, number of diagnoses

^b^based on Chi-square or Fisher’s exact test comparing difference in %. The data shows n (%) in each randomized group

^c^Logistic regression models failed to converge for Medicine Reconciliation and PCP F/u due to data sparsity, therefore, no odds ratio was estimated for the two outcomes

The sample consisted of 102 patients who fulfilled the eligibility criteria, were randomized to receive either TTOC or Standard of Care (Fig. 1). Randomization was conducted using a computer-generated 1:1 random number allocation sequence through REDCap [22]. Eligible patients were adult patients (≥ 30 years), with 2 or more chronic disease processes, English speakers, with good cognitive function, a life expectancy greater than 6 months, with an ability to provide consent. All patients were hospitalized at Stony Brook University Hospital and discharged to the patient home, with the follow up care in either the Family or Internal Medicine clinical practices. Patients self-identified as living within reasonable commute to the Family or Internal Medical Group clinics and were able to complete a technological aptitude test of turning on the telehealth technology and following the prompts. Patients were excluded if they had physical limitations prohibiting the use of the telehealth equipment, were uninsured (who received referrals elsewhere for follow up care), if involved in another research study, were pregnant or actively trying to conceive, or if admitted for a primary psychiatric diagnosis.

We conducted a 2-arm, parallel group, randomized controlled trial between June 1, 2017 to June 1, 2018. The standard of care for discharge planning, includes reviewing patient instructions and the discharge summary. The patient is encouraged to follow up with the PCP within 7–14 days and with scheduled specialist appointments as indicated [22]. The Telehealth intervention involved the provision of a telehealth kit, which included a smart phone device a Bluetooth-enabled blood pressure monitoring cuff, pulse oximeter, weighing scale, within 2 days after hospital discharge [22]. Telehealth patients measured their vitals daily using the tele-equipment and had weekly virtual visits with a transition of care physician (teledoc). During visits with the teledoc, medicine reconciliation was documented and medication adherence assessed in concordance with best practice from published studies [24] Physicians were trained to ask open-ended questions to assess medications taken, as well as perform validation of patient histories using Electronic Medical Record (EMR) and pharmacy data. Clinicians documented adherence if there were no discrepancies between physician prescription and patient self-report and data. Upon consent, patients participated in the trial for the length of thirty days following hospital discharge. All patients were consented and enrolled in the Health Information Exchange.

Study data was collected and managed using REDCap [25] electronic data capture tools hosted at Stony Brook Medicine. Primary Outcomes of hospital readmission and ED utilization were assessed using Health Information Exchange and EMR data. Secondary outcomes were assessed through phone surveys, whose data was hosted on REDCap. Medication adherence and reconciliation data was collected in REDCap during the virtual visit. For the control group, the EMR medicine reconciliation records and clinical notes were reviewed by a physician, for documented non-adherence to the discharge summary treatment plan.

Frequencies and percentages were calculated for categorical variables, e.g. Re-admission and ED visit for each randomized group. Means and standard deviations (SD) were calculated for continuous variables if the data followed a normal distribution (e.g. age). If the data did not follow the normal distribution, medians and ranges were calculated instead. Chi-square tests or Fisher’s exact tests were used to compare the percentages of categorical variables between the Telehealth and control groups. T tests or Wilcoxon rank sum tests were used to compared means and medians between the two randomized groups. To evaluate the associations between Readmission, ED visit and adhere with telehealth and other factors, multivariable logistic regression models were performed, and odds ratios and their 95% confidence intervals were estimated.

SAS v9.3 (Cary, NC) and SPSS v25 (Chicago, IL) were used to conduct all statistical analyses.

451 patients were assessed for eligibility for the trial. 102 patients met inclusion criteria for study participation, gave informed consent, and were enrolled in the study prior to discharge. 45 patients were randomized to the TTOC group while 57 patients received the standard of care. Patients were excluded if they no longer met the criteria of being discharged to home; if their hospitalization course worsened, requiring discharge to a subacute rehabilitation center (SAR). A small proportion of patients later refused to participate in the trial when discharged to the home (Fig. 1).

Overall, the study arms were balanced by characteristics as there were no statistically significant differences between the groups in regards to demographics including gender, race and education (Table 1). The average age at enrollment was 65 years. 68.5% of individuals in the control group had higher than high-school level of education in comparison to 76.1% of individuals in the telehealth intervention group. In addition, the employment rate was similar in both groups: 43.1% in the control group and 38.3% in the intervention group. No significant difference was seen in the presence of a computer at home: 77.6% for the control group and 63.8% for the telehealth intervention group. Over 97% of study participants had internet in their home, while over 94% owned a cell phone. Both groups had high percentages of patients who used a computer on a regular basis 63.8% in the control group and 77.6% of the TTOC group. Both groups showed high confidence in using smart phones or tablets. On average both groups spent about 4 h on the computer per day. Similar percentage of individuals in both groups reported their health as either good or very good: 60.3% in the control group and 48.9% in the intervention group. The median scores, on a questionnaire scale of 1–10, were also similar between the two groups for the confidence with health management and comfort with technology measures. Patients had an average of nine diagnoses, and a moderate severity risk score and a moderate rating of disability.

Patients had a statistically significant improvement in enthusiasm and confidence that Telehealth helped patients (*p* = 0.0001). There was no statistically significant difference in the perception of difficulty in participating in the trial (*p* > 0.072).

There was no statistically significant difference in follow up with the PCP (*p* > 0.096). However, 94% of patients in the Telehealth arm felt that the remote patient monitoring technology was helpful in managing their healthcare needs. 98% if given the opportunity would continue to use the technology to manage their health needs. 100% of the Telehealth patients found the intervention to be valuable. Also, Patients in the Telehealth arm, were about 7 times more likely to adhere to their medications (OR = 6.925, 95% CI: 1.2–39.9, *p* = 0.03).

There were no statistically significance regarding ED utilization or Hospital readmissions. Patients with a greater number of diagnoses were more likely to go to the ED (controlling for age, gender and Telehealth).

## Discussion

Our trial shows that when patients receive high quality tele-transitions of care, they are more adherent to their medications, and can be engaged in their healthcare. Telehealth provided great value for patients after hospital discharge. The trial was underpowered to evaluate hospital readmissions and ED utilization; however, it is important to recognize that telehealth patients received safe well-coordinated care for their medical conditions. There were no adverse events reported resulting from the Telehealth intervention in regards to patient injury, harm, error or death, despite patients suffering multiple co-morbidities and health changes. Four patients were successfully and actively referred to the hospital after having life threatening clinical situations including: stroke, acute airway and oxygen desaturation, reflecting their late-stage non-modifiable pathologies. Telehealth has shown promise in regards to reducing readmissions. However new literature also questions the validity of hospital readmissions as an endpoint, as some studies show increased death related to lower rates of readmission [26, 27]. The coordinated care of our telehealth patients in a setting with a shared EMR, also allowed for improved diagnosis, cohesive patient histories validated by clinicians with supportive bio-monitored data (blood pressure, heart rate, O2 saturation, weight).

There were other many benefits of this study, including the 18 residents formally trained in Telehealth, and resulting in 15 Primary care providers requesting formal credentialing for Telehealth from our institution in Family and Internal Medicine. The clinician engagement and training is an important achievement as physician adoption remains a considerable barrier to telehealth implementation [20].

This study lends itself to generalizability in academic medical systems; Stony Brook Medicine, is a large academic training hospital in which telehealth training is promoted for resident physicians. Smaller healthcare institutions without clinical trainees, may require additional efforts for clinician adoption. It is also important to note that clinical trainees were in their last year of training and therefore, the seniority of trainees involved in the role of the teledoc, should be taken into account. Furthermore, the volume of patients followed by the Teledoc must be tailored to clinical experience and aptitude. Further studies are needed to validate whether telehealth interventions using remote patient monitoring and video visits can reduce hospital readmissions and ED utilization.

As digital technologies become increasingly more important in patient’s lives and with the increased consumerism of both IT and healthcare itself, health care systems are faced with stronger demands for virtual health services. Our trial shows feasibility in implementing Telehealth within existing clinical workflows. TTOC has shown tremendous value for patients, clinicians and the hospital system, irrespective of being underpowered for readmissions and ED utilization. Further studies and large clinical trials in collaboration with several health systems, will allow for true return on investment for Telehealth for transitions of care.

## Conclusion

Telehealth has great value in providing safe transitions of care, increasing patient satisfaction and improving patient adherence to medication. More research is needed to evaluate the true impact of Telehealth on preventing avoidable hospital readmission and ED visits.

## Data Availability

The datasets used and/or analyzed during the current study are available from the corresponding author on reasonable request.

## Abbreviations

ED: Emergency Department
PCP: Primary care provider
SAR: Subacute rehabilitation centers
SD: Standard deviations
teledoc: Telehealth transition of care physician
TTOC: Tele-Transitions of Care
